# Elemental and Thermo-gravimetric Characterization of Trace Metals in Leaves and Soils as Bioindicators of Pollution in Kyiv City

**DOI:** 10.1007/s11270-021-05277-y

**Published:** 2021-08-09

**Authors:** Mohamed Tarik, Michael Hoffmann, Sergey Shmarin, Ajay Bhagwan Patil, Christian Ludwig

**Affiliations:** 1 Bioenergy and Catalysis Laboratory (LBK), Energy and Environment Research Division (ENE), Paul Scherrer Institut (PSI), CH 5232 Villigen PSI, Switzerland; 2 Institute of Water Problems and Land Reclamation of the National Academy of Agrarian Sciences of Ukraine, Kyiv, Ukraine; 3 National Power Company Ukrenergo, Kyiv, Ukraine; 4 Environmental Engineering Institute (IIE), School of Architecture, Civil and Environmental Engineering (ENAC), École Polytechnique Fédérale de Lausanne (EPFL), CH 1015 Lausanne, Switzerland

**Keywords:** Trace metals, TGA-ICP-OES, Environmental biomarkers, Metal volatilization

## Abstract

In this study, leaf and soil samples were used as bio-monitors for different alkali and heavy metals at six different locations in Kyiv city. Using x–y plots of the inductively coupled plasma optical emission spectroscopy (ICP-OES) data measured the discrepancy level in elemental composition between the different investigated areas; the correlation between the concentrations in tree leaves and the samples from the surrounding soils were investigated. While the concentration of essential mineral elements and metals was found to be similar in several leaf and soil samples, in other samples, their concentration spread up to more than one order of magnitude. The concentration of metals was found to be higher in soil samples than in leaves. Thermo-gravimetric analysis (TGA) data helped to further characterize both types of samples. The metal removal during the incineration of the leaves was investigated by coupling a thermo-gravimetric analyzer to an inductively coupled plasma optical emission spectrometer (TGA-ICP-OES). The release of Cd, K, Na, Pb, and Zn during incineration at temperatures up to 960 °C was online monitored, and some insights were drawn about the behavior of such metals and the chemistry involved in the volatilization process.

## Introduction

Plants and soils in urban areas can be used as bio-monitors for pollution. In many studies, tree leaves were used to monitor the soil and air pollution of specific areas by metals and toxic heavy metals (Aboal et al., 2004; Bekuzarova et al., 2017; De Nicola et al., 2008; P. Madejón et al., 2013; Paula Madejón et al., 2006; Wuytack et al., 2010). Tree leaves are good accumulators of particle dust and hence an excellent bio-monitor. Trapping fine atmospheric particles by plants can have positive effects on reducing risks to the environment and human health. Air contamination of urban areas compared to rural ones can be quantified by analyzing unwashed leaves. In this case, the concentrations of some elements might be higher than the natural background, because of the disposition from the surrounding atmosphere or the selective uptake through the tree roots. Among others, the contamination level by metals depends on the tree type and its location. The interaction of a tree with the surrounding soil (e.g., elements uptake through tree roots) and atmospheric contaminations (e.g., caused by industrial pollution sources) play a crucial role in the contamination level of the leaves by metals and heavy metals. For instance, the use of lead gasoline in the past is a good example of such heavy metal contamination. Ram et al. reviewed the recent scientific studies investigating the ability of plants to remove pollutants from air (including gaseous and airborne dust) and their essential role in carbon dioxide sequestration and oxygen release (Ram et al., 2015). In an assessment study, the leaves and bark were used as bioindicators to monitor the heavy metals (Cd, Cu, Pb, and Zn) pollution in urban areas in the city of Katowice (Poland). Compared to background values, it was reported that only bark samples were reflecting the pollution level (Steindor et al., 2016).

The composition of soils is affected by the geological properties, the surrounding flora and fauna, and anthropogenic sources such as industrial plants. The contribution of each of them is challenging to be separately quantified mainly in short-term investigations. In particular, heavy metals in soils can be originated from natural sources (e.g., because of weathering) or anthropogenic sources such as the application of sewage sludge, fertilizer, and pesticides. Recently, a review was published about the contamination by metals in the region close to Aznalcóllar mine (Spain), the soil–plant relationship, and the possible contamination transfer to plants. The authors reported the efforts done in the last 20 years to understand the trace elements dynamics in this contaminated area and the work done up to now to decontaminate it (Paula Madejón et al., 2018). Sun et al. monitored the level of sulfur and heavy metals in an urban–rural gradient in Guangzhou (China) by analyzing needles and soils of Masson pine trees (Sun et al., 2009). To monitor Cd, Hg, and Pb pollution level in five sites in the city of Brno (Czech Republic), Kleckerová et al. analyzed dandelion plants and soil samples. The highest contaminated area was one with high traffic density, and a good correlation of these metals in leaves and related soils was found (Kleckerová & Dočekalová, 2014). Zhai et al. investigated the pollution along highways in Hunan Province (China) caused by heavy metals in roadside soils and it was found that Cd, Pb, and Zn were available for uptake by wild plants in this area and Cd was presenting the significant pollution factor (Zhu et al., 2016).

In order to remove heavy metals, several soil treatment methods exist, including leaching, washing, phytoextraction, electro-kinetic remediation, and thermal treatment (Liu et al., 2018; Rivero-Huguet & Marshall, 2011; Suzuki et al., 2014; Wu et al., 2012). Particular types of plants, known as hyper-accumulators, can store a large amount of metals and heavy metals and hence can be used for the remediation of contaminated soils (Ali et al., 2013; Guo et al., 2018; Muthusaravanan et al., 2018; Sheoran et al., 2016; Tauqeer et al., 2016; Wan et al., 2016). These plants can be incinerated to remove/stabilize the heavy metals (Connor et al., 2018; Keller et al., 2005).

In this work, we report about the monitoring of different trace elements in six different sites in Kyiv city (Ukraine) by using inductively coupled plasma optical emission spectroscopy (ICP-OES). Using simple x–y plots, a straightforward comparison is made between the plants among each other and between the plants and the related soils. The thermal treatment of the leaves as waste material was studied using a hyphenated technique by coupling a thermo-gravimetric analyzer to the ICP-OES instrument. Using this online setup, the release of two alkali metals (Na, K) and three heavy metals (Cd, Zn, and Pb) was investigated.

## Experimental Section

### Sample Preparation

In this work, 18 samples (6 leaf and 12 soil samples) were collected from 6 different urban areas of Kyiv city at the end of the autumn and packed in polyethene bags. From each location, two soil samples were taken: one close to the corresponding tree (labeled as soil (a)) and one 10 m apart (labeled as soil (b)). All samples, except sample 6 (leaf and soil samples), were collected in the city center. Collection place 6 is located in the city periphery at about 8 km from the center. The collected leaves are from four different types of trees, namely goat willow, maple, pear, and oak. The sampling locations of samples 1, 2, and 4 were close to the traffic zones, while samples 3 and 5 were nearby a gas station and a combined heat and power plant, respectively. In this work, a no-wash procedure was applied to the leaves in order to preserve any airborne potential contamination on them. The leaves were first dried; then, after adding liquid nitrogen, the samples were ground manually by ceramic mortar and pestle.

### Elemental Analysis

All samples were dissolved in different concentrated acids. For leaves, about 150 mg of prepared sample was dissolved in 2 mL H_2_O_2_ (50%), 3 mL of HNO_3_ (65%), 2 mL HCl (30%), and 2 mL HF (50%). To about 200 mg of each soil sample, 6 mL HNO_3_, 2 mL HCl, and 3 mL HF were added. All used acids were high-purity acids (from Sigma-Aldrich). All samples were then digested in a high-pressure microwave digestion unit (Multiwave 3000, Anton Paar, Austria). The digested samples were afterwards diluted in Milli-Q water (18.2 MΩ·cm) with a total dilution factor between 170 and 250. The quantification of 34 elements was made by using external standard calibration. Blank solutions were prepared using the 1% HNO_3_ solution. The standards were prepared by diluting multi-element standard solutions (Bernd Kraft, Germany) in 1% HNO_3_ down to a range of concentrations between 0 and 10 μg/mL. A 1 ppm standard solution was used as QC standards. Because of the use of the quartz ICP introduction system and the use of HF, the measured Si concentrations in soils were found to be overestimated, and hence, the related data were excluded. After the measurement of a set of samples and at the end of each measurement, the blank solution and at least two standard solutions were measured, to check for any drift or signal suppression. The elemental analysis (two replicates for each sample) was carried out by using an ICP-OES instrument (Spectro Arcos, Germany).

### TGA and TGA-ICP-OES Analysis

All leaf and soil samples were analyzed using a thermo-gravimetric analyzer (TGA/DSC, Mettler Toledo, Switzerland). In each measurement, a weight of about 200 mg of each leaf or 400 mg of each soil sample was treated at a time. The thermal treatment of all samples was performed under oxidizing conditions by using O_2_/Ar (20/80% v/v) gas mixture with a total flow of 100 mL/min. The temperature program was set as follows: first, the samples were heated up to 105 °C and kept at this temperature for 10 min, then the temperature was increased up to 580 °C with a heating rate of 10 °C/min, then with 6 °C/min up to 960 °C, and finally, it was maintained at 960 °C for 30 min.

To investigate the evaporation behavior of 2 alkali metals (K and Na) and 3 heavy metals (Cd, Pb, and Zn), the leaves were thermally treated and were analyzed online. For this purpose, the same TGA was coupled with the ICP-OES system (TGA-ICP-OES) as described by Ludwig et al. (2007). This hyphenated setup allowed simultaneously thermo-gravimetric and elemental analysis. The thermal treatment program was the same for all TGA experiments. To further dilute the generated aerosol and to avoid condensation of evaporated material, 500 mL/min argon carrier gas was added directly after the outlet of the TGA. Beside the low amount generated at low temperatures, the resulted aerosol introduced into the plasma was completely dry and no specific changes were needed in operating parameters.

## Results and Discussion

### Elemental composition of the leaf and soil samples

**Fig. 1:**
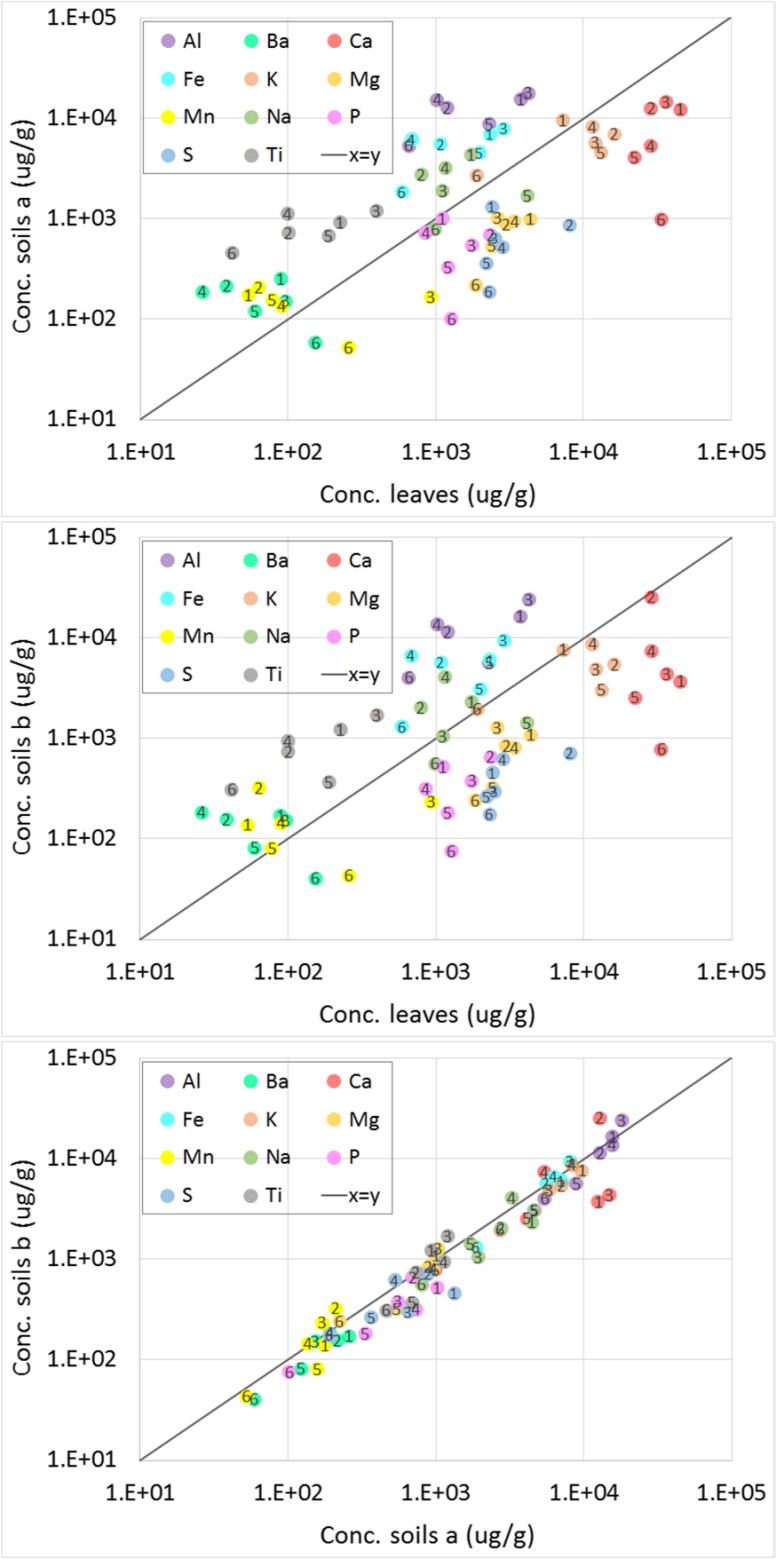
Correlation diagrams of the major and minor elements in leaf and soil samples having a concentration from 10 μg/g to 10 wt %. The numbers 1 to 6 refer to the different investigated zones in Kyiv city

**Fig. 2:**
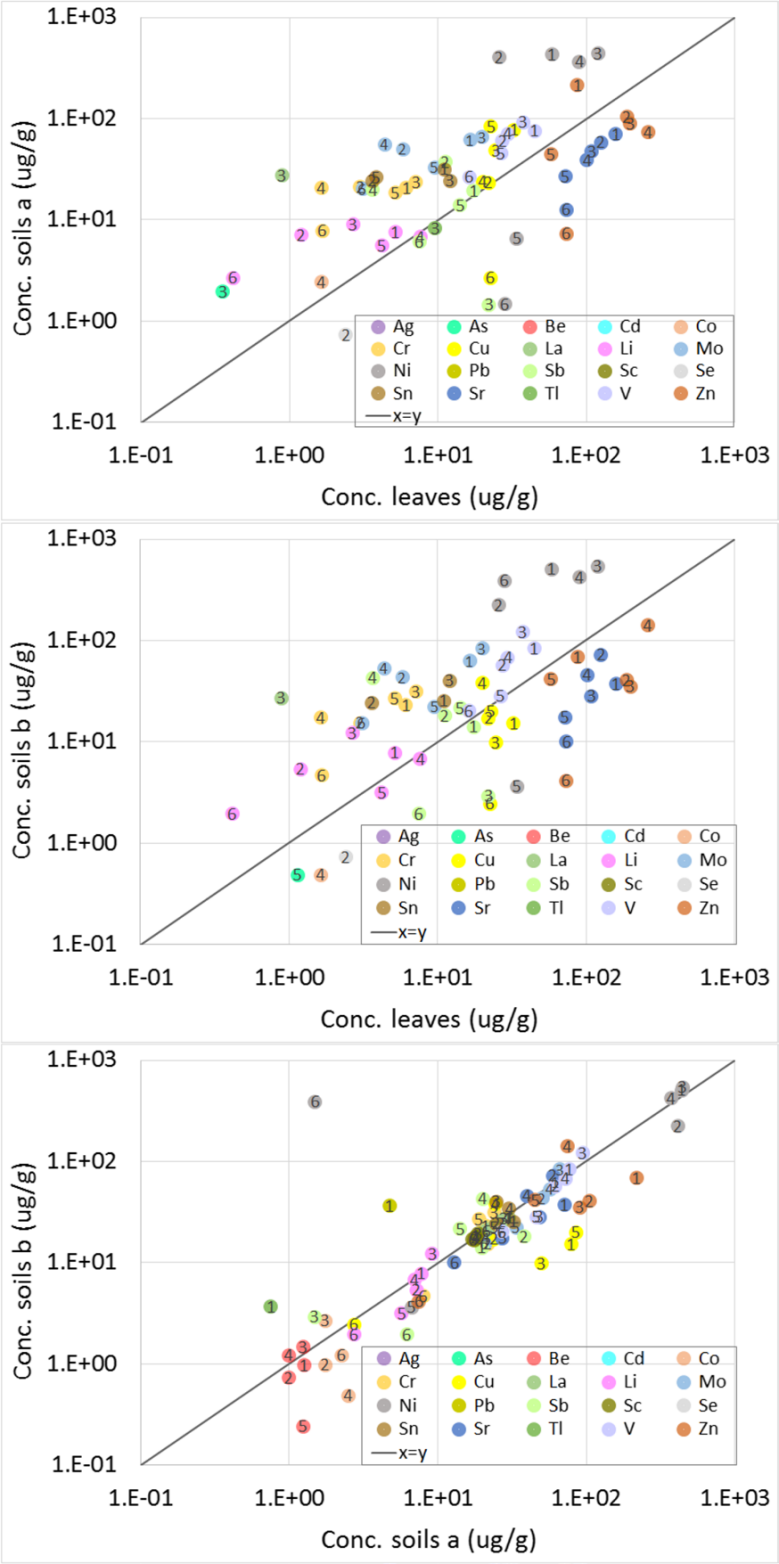
Correlation diagrams of the trace elements in leaf and soil samples having a concentration from 0.1 to 1000 μg/g. The numbers 1 to 6 refer to the different investigated zones in Kyiv city

In order to compare the elemental compositions of the different collection areas and to check for a correlation between the composition of the leaves and the corresponding soil samples, the concentration of the measured elements (major, minor, and trace elements) in soils (a) and (b) is plotted in the logarithmic scale against that of the leaves (Figs. 1 and 2). In the same figures, the concentration of the elements in the different soil samples (b) is also plotted versus the soil samples (a). The elements which have one or more concentration value(s) below the quantification limits (based on the 10 times the standard deviation of the blank) are not plotted. All reported concentrations have a relative standard deviation below 5%.

#### Elemental Composition of the Leaves

Comparing the average concentrations of the elements measured in the leaves (Figs. 1 and 2), the following can be concluded: the average concentration of abundant elements (from few wt % down to 0.1%) was in the order Ca > K > Mg > Al > S > Fe > Na > P, while that of the main trace elements (average concentration from 100 to 1000 μg/g) was found as follows: Ti > Mn > Zn > Sr > Ba > Ni > V > Cu > Sb > Pb > Mo > Ag. Low average concentrations (< 10 μg/g) were in the order Sn > Se > Tl > Cr > Li. Lowest traces (close or lower than the detection limit of ICP-OES) were measured in leaves for As, La, Cd, and Co.

Comparing the concentrations of the leaves among each other, the major elements Ca, K (except for leaf 6), S (except for leaf 2), Mg, Na (except for leaf 5), and P have a narrow data spread in all leaves (Fig. 1). For these essential elements, the geological properties might be similar for all locations investigated in this study. More variation was found for the abundant elements Al, Fe, and Ti, indicating possible effects of natural or anthropogenic sources. The concentration of all trace elements in leaves shows a spread of factor between 2 and 10, besides that of Cu, which had a narrow spread (Fig. 2). These trace elements seem to be more affected by either atmospheric deposition or by uptake from the soil. The sample of leaf 3 has the maximum concentration of Cr, Mg, Mo, and Ni, and leaf 6 has the minimum concentration of Cr, Mo, and V.

#### Elemental Composition in the Soil Samples

The concentrations of the major elements (in average and excluding Si) in soil samples were found in the order Al > Ca > K > Fe > Na > Ti > Mg > S > P (Fig. 1). The trace elements with relatively high concentrations were found in the following order: Ni > Ba > Mn > Zn > V > Mo > Cu > Sr > Sn > La > Cr > Sc > Sb (Fig. 2). The low trace elements were measured in the order: Li > Pb > Tl > As > Co > Se. For soil (b), a similar trend to that of soil (a) was observed except Fe > K, Mn > Ba, V > Zn, Pb > Sr, and Cu > Sb. From the analysis data, the average concentrations of all expected macro- and primary, secondary, and tertiary micro-nutrients are found in expected level in different leaves and soils investigated. This is most probably related to the mineralogical properties of soils. However, the concentration of some heavy metals is reaching the soil contamination level reported in the literature (Vodyanitskii, 2016).

At location 6 for all envisaged elements, the concentration was lower in all soil samples, except that of Sb and Ni which were measured in soil 3a and soil 5b, respectively. Excluding soil sample 6, the spread of the concentrations of Al, Ba, Fe, K, Mg, Mn, Na, and Ti is narrow. The concentration of the other major elements varies by less than or about 1 order of magnitude. Excluding again sample 6, the concentrations of the trace elements Cr, Li, Mo, Ni (except the sample 5), and Sn have also a narrow spread, while the concentration range of the other traces varied by less than or about one order of magnitude. For the metals and heavy metals in the soil sample, sample 3 (a and b) has higher concentrations of Cr, Mo, Ni, and V while the maximum concentration of Cu, Sb, and Zn was measured in the soil sample 4. Similar correlations for heavy metals (Cu, Cd, Zn, etc.) were observed in urban environment indicating the effect of anthropogenic activities (Kleckerová & Dočekalová, 2014; Steindor et al., 2016; Zhu et al., 2016). Again the narrow spread of the concentrations of many nutrients can be related to the mineralogical properties of soils. However, the lowest concentration of several elements was found in location 6, revealing that the other soil samples might be substantially affected by pollution (e.g., due to traffic and industrial activities). This is the case, for example, for Cu and Ni. Pollution effects can also explain the higher level of As, La, and Tl in the leaf and the soil samples from location 3, Co from location 4, Zn in soil 1 and leaf 4, and Sr for all samples except sample 6.

#### Leaf-Soil Comparison

Correlating the element concentrations in the leaves to that of the related soil samples, it can be deduced that, in most cases, the major elements Ca, K, S, Mg, and P are enriched in leaves. At the same time, Al, Ba, Fe, Na, and Ti are higher in the soil samples (Fig. 1). For the trace elements (Fig. 2), Sr and Zn (except in soil 1 (a)) have concentrations higher in the leaves than that of soil samples, while the rest of the traces are mostly higher in the soil samples. Cu and Sb showed higher concentration in leaves 3 and 6, respectively. The elements present in higher concentration in the soil samples than in the leaves can be mainly related to the mineralogical properties of these areas, while the ones higher in the leaves might be caused by atmospheric deposition or by uptake and accumulation. This is the case for the metals mentioned above Cu, Sb, Sr, and Zn.

#### Soil-Soil Comparison

Comparing the soil samples among each other, although the relationship between the concentrations of several major elements can be roughly presented by the equation x ~ y (Fig. 1), some of them (mostly nutrient elements) are slightly more enriched in soil (a) than in soil (b). This small discrepancy cannot be explained. Larger spread and higher concentration in soil (a) was found for the trace elements Cu, Co, and Zn. The other trace elements are quite similar in both soil samples (a) and (b) (Fig. 2). Lower Ni concentration was measured in sample 6, indicating most probably that this element is profoundly affected by anthropogenic pollution. The similarity between soil (a) and the related soil (b) ((b) is 10 m far from (a)) and the small spread of the concentration in the different soils indicate that there is no difference in the pollution pattern for the locations investigated, except for Cu, Co, and Zn.

### Thermal treatment of the leaf and soil samples

**Fig. 3:**
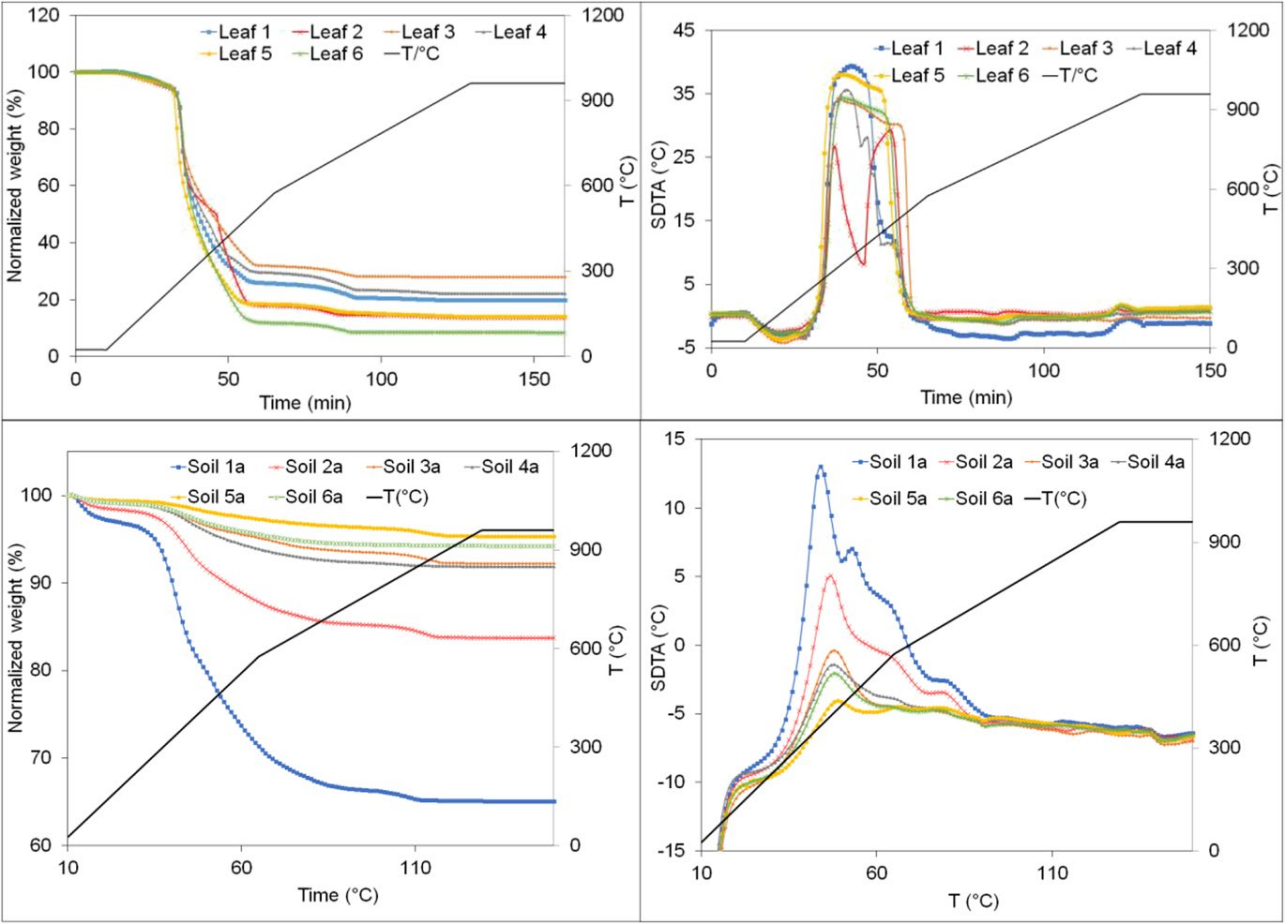
Thermo-gravimetric data (weight loss (in %) and DTA (in °C)) as a function of the temperature of leaf and soil sample (a)

Further insights about the samples were gained by TGA analysis. In Fig. 3, the TGA weight loss (in %) is plotted as a function of temperature for the 6 leaf and soil samples (a), respectively. The thermo-gravimetric behavior is similar in the same kind of samples (leaf or soil samples). The part of the curve corresponding to weight loss for the leaves up to 200 °C is related to the removal of the remaining moisture. The difference in the organic content, which evaporate totally at moderate temperatures (from 220 to about to 650 °C), leads to a significant difference in the total percentage of the evaporated and the solid residues of the 6 samples for both leaves as well as soil samples. The total weight loss of leaves due to the evaporation of inorganic materials (< 700 °C), including metal compounds, ranges from 2 to 7%. The solid residue of leaf 6 had the lowest weight.

The soil samples (a) also showed similar behavior. However, the evaporation (of water and organic compounds) up to 650 °C was found to be higher in samples 1 and 2. The evaporated inorganic material at higher temperatures was higher in soil samples 2 and 4 (~ 2%) and less in samples 1, 3, and 5 (~ 1%). The minimal volatilization of organic and inorganic materials was observed for soil sample 6 (~ 5% and < 0.1%, respectively). These data show that the organic content is quite different and mainly high in soil samples 1 and 2 and the inorganic evaporation was somewhat high in all samples except in sample 6. For the leaves, the evaporation of the inorganic part is minimal due to its lower content and the limited volume allowed by the TGA crucibles.

The DTA (differential thermal analysis) of leaves are plotted in Fig. 3. The shape of all curves of leaves is quite similar, except that of sample 2, which showed 2 exothermal peaks. For each leaf, a broad exothermic peak was observed in the ranges between 320 and 380 °C. At a temperature of about 880 °C, the DTA curves of all leaves show a small exothermal peak. For all soil samples, a large peak was detected at about 300 and 340 °C. Between 350 and 590 °C, the curves showed a two-step plateau and a small drop at about 880 °C. These data show that the elemental composition of the different samples has possibly more effect on the incineration of the organic compounds of the leaves compared to that of the soil samples. The evaporation of inorganics at high temperatures has similar behavior in all samples (in leaf as well as in soil samples).

**Fig. 4:**
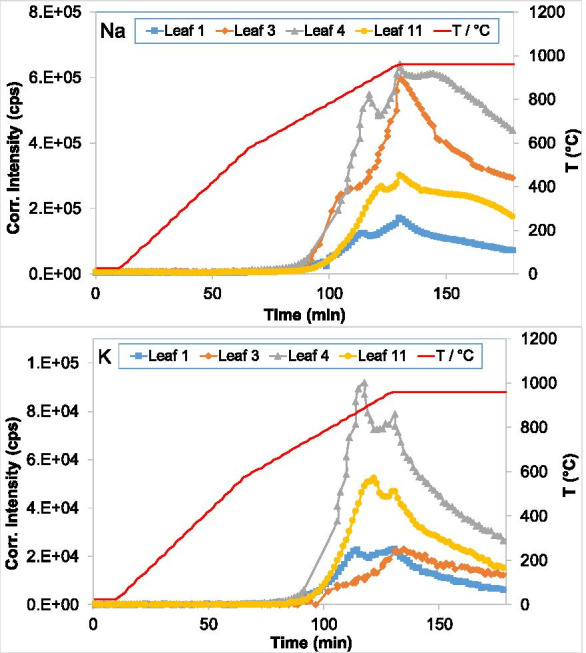
ICP signals of evaporated Na and K as a function of temperature during the thermally treated leaves

**Fig. 5:**
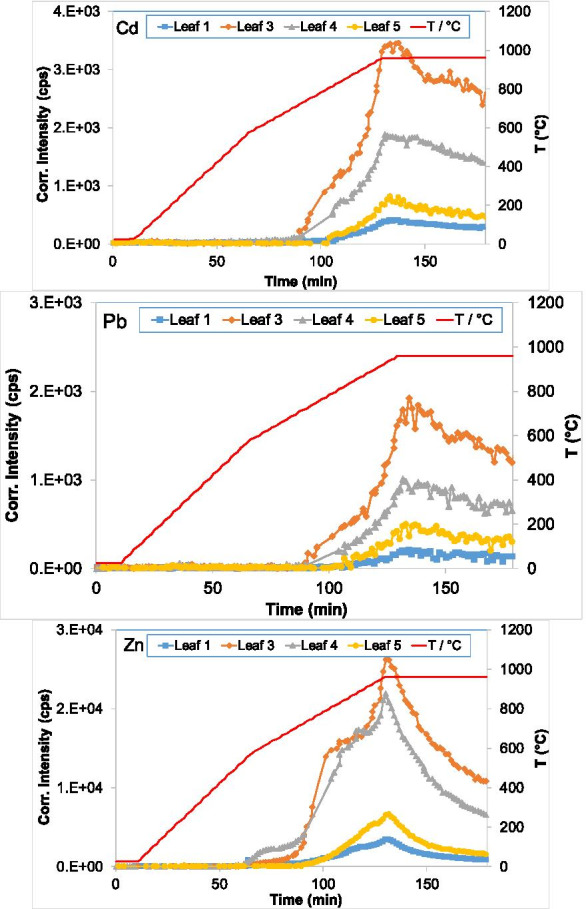
ICP signals of evaporated Cd, Pb, and Zn as a function of temperature during the thermally treated leaves

The incineration can be used to remove or stabilize the hazardous metals in the leaves. Therefore, the dried leaf samples were thermally treated under the same oxidizing conditions (100 mL Ar/O_2_ mixture, 20% O_2_ v/v). The combination of TGA and ICP-OES instruments for online measurements provides a unique opportunity to acquire simultaneously thermo-gravimetric and elemental information. Two major elements (Na and K) and three heavy metals (Cd, Pb, and Zn) were measured by ICP-OES. In Figs. 4 and 5, the ICP signals of Na, K, Cd, Pb, and Zn are plotted as a function of time during the thermal treatment of leaves. The temperature program is depicted on the second y-axis. To accurately compare the emission in the different samples and because different weights are used in the TGA-ICP measurements, the ICP signal intensities are normalized to a starting weight of 200 mg. The evaporation of Na, K, Cd, Pb, and Zn starts at about 665, 665, 720, 725, and 620 °C, respectively, and shows slight changes between the different leaves. Na and K show two maxima: one slightly before the isothermal regime at 960 °C and one after. This latter coincides with the maximum detected for all three heavy metals. All signals of the five elements decreased afterwards. The signals of Na and K in all leaves decrease in the following order: 4 > 3 > 5 > 1 and 4 > 5 > 1 > 3, respectively, while that of all three metals in the same order: 3 > 4 > 5 > 1. The TGA-ICP-OES data show that the emission of alkali metals has two peaks behavior. The second peak coincides with the peak detected for the three metals measured (Cd, Pb, and Zn). These results show that the metals are most probably emitted as chloride after reacting with Cl available as alkali salts. For high contaminated soils and biomass materials, the addition of chlorine compounds can boost their release utilizing thermal treatment (Jakob et al., 1995, 1996). However, the matrix and locally generated redox conditions can also affect the release of these metals. For Cd and Zn (having a boiling point of 765 °C and 907 °C, respectively), it is possible that they can volatilize in elemental form and re-form oxides by interacting with O_2_ (Foppiano et al., 2018). A linear function can approximately fit the decrease of the metal signals during the isothermal regime (at 960 °C). This behavior might be related to the decrease of the reactants (including the metal and the alkali content) and the kinetics of the corresponding reaction. A linear extrapolation of the metal signal in leaf 3 suggested that total decontamination can be reached for Cd, Pb, and Zn after about 305, 286, and 236 min, respectively, under the given conditions of this study.

### Statistical analysis for the anthropogenic effects or sample differences

As mentioned in the earlier section, the samples differ in locations (6 locations including collected in the city center (more exposure to pollution) and away from the city center (less exposure to pollution) and sample types such as leaves and soils (two types) from earlier mentioned location). With respect to 18 sampling scenarios (measured for each element under study) for the effect of pollution, there are clear trends, evident from the ICP-OES measurements and TGA-ICP-OES studies showing the anthropogenic effect on the samples taken inside the city. Similar effects of anthropogenic activities were observed for metals Cu, Cd, and Zn, in literature (Kleckerová & Dočekalová, 2014; Steindor et al., 2016; Zhu et al., 2016).

Our approach was very systematic with respect to versatile sampling (representing center, power station, and suburban areas to correlate the data), replicate measurements, and correlation. Different leaves vs soils and soil vs soil x–y plots clearly show the narrow fit and hence the correlation. The trend helps to understand that the similar plant/soil samples have less loading of heavy metals in the sample (6) taken away from the city center and that there is a role of anthropogenic factor. Also evident from the TGA-ICP-OES studies is that the minimum organic and inorganics lost in these samples. Even though the mechanistic aspects regarding the atmospheric deposition or by uptake are beyond the scope of present work, additional multivariate analysis was done to show that these effects are statistically significant in a given set of samples and experimental conditions. Table 1 clearly shows that Cr, La, Mo, Sr, and V have significant differences in the concentration with respect to sample locations and type (multivariate analysis with ANOVA: two-factor without replication). Therefore, the effect of the anthropogenic factors is evident.

**Table 1:** ANOVA: two-factor without replication analysis of ICP-OES data

No	Metals	Type of effect	df	*F*	*P*-value	Fcrit	Significant anthropogenic effect vs location/sample type
1	***Co***	Sample location	5	0.7081	0.6308	3.3258	No significant effect or difference
		Sample type	2	2.5238	0.1296	4.1028	*F* < Fcrit; *P* > 0.05
2	***Cr***	Sample location	5	4.5723	0.0198	3.3258	**Significant effect or difference**
		Sample type	2	24.2524	0.0002	4.1028	*F* > Fcrit; *P* < 0.05
3	***Cu***	Sample location	5	1.2800	0.3449	3.3258	No significant effect or difference
		Sample type	2	3.0192	0.0942	4.1028	*F* < Fcrit; *P* > 0.05
4	***La***	Sample location	5	4.1637	0.0263	3.3258	**Significant effect or difference**
		Sample type	2	397.4699	2.96E-10	4.1028	*F* > Fcrit; *P* < 0.05
5	***Mo***	Sample location	5	6.2831	0.0069	3.3258	**Significant effect or difference**
		Sample type	2	23.3866	0.0002	4.1028	*F* > Fcrit; *P* < 0.05
6	***Pb***	Sample location	5	0.9823	0.4740	3.3258	No significant effect or difference
		Sample type	2	0.4836	0.6303	4.1028	*F* < Fcrit; *P* > 0.05
7	***Sb***	Sample location	5	1.042	0.4446	3.3258	No significant effect or difference
		Sample type	2	0.2573	0.7781	4.1028	*F* < Fcrit; *P* > 0.05
8	***Sr***	Sample location	5	8.3609	0.0024	3.3258	**Significant effect or difference**
		Sample type	2	46.0388	9.02E-06	4.1028	*F* > Fcrit; *P* < 0.05
9	***V***	Sample location	5	6.7105	0.0055	3.3258	**Significant effect or difference**
		Sample type	2	9.3129	0.0052	4.1028	*F* > Fcrit; *P* < 0.05
10	***Zn***	Sample location	5	2.0979	0.1492	3.3258	No significant effect or difference
		Sample type	2	3.3647	0.0763	4.1028	*F* < Fcrit; *P* > 0.05

Boldface indicates the metal dataset with significant anthropogenic effect as described in section 3.3

## Conclusions

In this work, the elemental composition of leaf and soil samples from 6 different locations in Kyiv was determined and presented in x–y plots, in order to monitor the correlation level between the composition of leaves and the corresponding soil samples and to find out whether a sampling location underwent potential contamination with a specific metal or not. By comparing the concentration of a specific element in the soil and the leaf samples from two different collection areas, the spread of the concentrations and their contamination level can be monitored. For the investigated samples, there is clear evidence of the uptake and/or airborne deposition of several metals in leaves (such as Cu, Sb, Sr, and Zn). Sample 6 taken far from the center shows the lowest concentrations of different elements, especially metals. This indicates the role of the anthropogenic effect (e.g., traffic) on the contamination of soil and trees in Kyiv’s city center. Moreover, the obtained data can be used as a reference database for the elemental composition of soils in the studied region.

Furthermore, thermal incineration might be an excellent way to treat/decontaminate contaminated biomass materials. The matrix and additives can play a crucial role in this process. Using TGA-ICP-OES as an online technique helps to track the emission of the metals of interest, to reveal their behavior during the thermal treatment, and to try to understand the chemistry involved in that. However, a mechanistic understanding is beyond the aspect of this study. The application of the TGA-ICP-OES hyphenated technique for heavy metals monitoring and treatment applications has been successfully demonstrated in the urban environment.

## Data Availability

The datasets generated during and/or analyzed during the current study are available from the corresponding author on reasonable request.
